# The interplay between genetic and lifestyle obesity-related risk factors could be an important reason for the increasing epidemic of diabetes mellitus

**DOI:** 10.3389/fendo.2026.1805010

**Published:** 2026-06-19

**Authors:** Salah Gariballa, Ghada S. M. Al-Bluwi, Javed Yasin

**Affiliations:** Internal Medicine, College of Medicine & Health Sciences, United Arab Emirates University, Al Ain, United Arab Emirates

**Keywords:** body weight, diabetes, genes, obesity, physical activity

## Abstract

**Introduction:**

Obesity-related type 2 diabetes (DM) is increasing rapidly and at present reaching epidemic proportions in some Worldwide populations. The aim of this study was to provide new knowledge on the interplay between genetic and lifestyle obesity-related risk factors in populations with the highest incidence of obesity-related diabetes could provide an important tool to help prevent or delay diabetes onset in high-risk groups.

**Methods:**

Community free-living individuals visiting primary health centers were recruited to the study following informed written consent. Demographic and clinical characteristics, physical activity, dietary intake and biological markers of DM were measured at baseline and follow up. Validated questionnaires were used to assess physical activity and dietary intakes. A Cox proportional hazards analysis was used to examine the risk of developing diabetes diagnosed using the WHO cut-of-points criterion of HbA1c ≥ 6.5% at follow after adjusting for known clinical risk indicators.

**Results:**

A total of 375 Community free-living locals UAE citizens subjects, 348 (93%) of them females and 253 non-locals’ expatriates [187 (73%)] females were recruited and followed up for a period of 427 ± 223 days. Using WHO cut-of-points for diagnosing DM (HbA1c ≥ 6.5%), 31 (6%) subjects out of 545 followed up developed DM. Overall local United Arab Emirates (UAE) citizens reported significantly lower levels of physical activity in comparison to non-local expatriates. The Cox proportional hazard model analysis revealed that being obese, UAE national and physically inactive is associated with a significantly increased risk of DM after adjusting for other prognostic indicators [non-UAE national: Odd ratio (95% CI): 0.13 (0.04, 0.47); p=0.002; physically active: 0.31 (0.11, 0.90); p=0.002]. The Kaplan Meier figures show the significantly increased risk of developing DM in physically inactive local UAE citizens compared with expatriates’ residents (p<0.05). In contrast risk of developing DM was no different between physically very active UAE nationals compared to non-nationals at follow up (P>0.5).

**Conclusion:**

Our finding suggests that physical inactivity in high-risk groups is the most important risk factor for developing DM. Urgent actions are needed to increase physical activity in this high-risk group coupled with further research to understand the reasons for this striking indigenous population variability.

## Background

A recent landmark paper on Worldwide trends in diabetes prevalence from 1990 to 2022 reported an estimated 828 million adults had diabetes, an increase of 630 million with the largest increase in South Asia, the Middle East and North Africa (1). If this trend continue health care systems will not be able to cope and treatment of diabetes and related complications will be prohibitively expensive and have adverse economic consequences because it is mainly affecting young and middle age working men (1–5). In the Gulf region including the United Arab Emirates (UAE) the prevalence of obesity and related type 2 diabetes is increasing relentlessly and reaching epidemic proportions at present (1–5). As result in the UAE, we have the 2^nd^ highest prevalence of obesity-related diabetes in the World (4, 5).

UAE society has been through recent and rapid socioeconomic changes over the last 50 years. Associated changes in diet and lifestyle are therefore thought to be the main drivers for the growing epidemic of overweight/obesity, type 2 diabetes and other related CVDs. Interactions between genes and environmental factors as a result of lifestyle changes in a relatively short period of time in UAE citizens compared to other nations may also have led to a uniquely different interplay of CVD risk factors in the UAE society, hence the higher rates of obesity and related diabetes compared to other populations (3, 5, 6). In other words, increased genetic susceptibility of UAE citizens to the adverse effects of certain risk factors such as obesity and physical inactivity may have led to a greater propensity for developing type 2 diabetes and other related CVDs compared with the Western populations (1, 3, 7). This partly explains the reasons why the CVD has been falling over the last 30 years in the USA and Europe despite increasing adult obesity, and only modest reductions in classical lifestyle-related risk factors. Despite the increase in obesity and related type 2 diabetes at this alarming rate in the UAE and other similar nations, surprisingly the molecular mechanisms and the magnitude of the contribution of individual modifiable risk factors to the increased risk of type 2 diabetes is not very clear (2, 8). The aim of this study was to identify important risk factors unique to the UAE citizens compared to non-UAE expatriate residents that are responsible for the relentless increase in diabetes epidemic in UAE citizens.

## Materials and methods

The sample of this study includes free-living Emirati (UAE citizens) and expatriates from other non-Gulf Arab countries aged 18 years and over. Community free living subjects were approached and invited to take part in the study. subjects had clinical, dietary, physical activity assessment and anthropometric measurements following an informed written consent to take part in the survey. A fasting blood sample was obtained for measurements of biological outcome measures including lipid profile, glycemic control marker, inflammatory markers, and other related clinical, nutritional variables. Individuals with severe chronic clinical or psychiatric disease, participating in other intervention trials, on dietary supplements or taking anti-obesity medications and those unable to give an informed written consent were excluded. The local research ethical committee has approved the study.

### Measurements

All participants had baseline clinical assessment including demographic and medical data, history of chronic illnesses, smoking, alcohol and drug intake. Anthropometric data including body weight, body mass index (BMI), fat mass and fat-free muscle mass were measured by bioelectrical impedance method using a standard validated Tanita M10T6360 analyzer. Results are shown on an easy-to-read display screen and printed on a sheet.

### Measurement of physical activity and dietary intakes

A validated questionnaire was used to assess occupation and leisure-related physical activity and also sedentary behavior. Data were obtained on frequency and duration of daily or weekly physical activity sessions for at least 20 minutes or more in which subjects became breathless or sweating. Questions were also asked about the number of hours subjects spend doing housework (9, 10). A validated short semi-quantitative food frequency questionnaire designed for self-administration following a brief verbal discussion was used to assess subject’s fruit and vegetables intake. It specifies the usual frequency of consumption of food items during the previous 12 months and assesses the average weekly nutrient consumption of each individual (11). Calorie intake measured in a subgroup of subjects using a locally validated 24-hour recalls once at baseline assessment and once at follow up visits.

#### Blood samples

Details of measurement of metabolic risk factors were published before (10). Briefly, fasting blood samples were drawn into 2 vacutainer tubes, containing potassium EDTA as anticoagulant. The samples were thoroughly mixed at room temperature and immediately transferred to the laboratory. Both tubes were centrifuged immediately for 10 min at 4000 rotations/min. Plasma and serum were collected and stored at -80 °C for future determinations of biochemical outcome measures. Circulating levels of renal and liver functions, lipids and high sensitivity C reactive protein (hs-CRP) were measured using an automated analyzer Integra 400 Plus (Roche Diagnostics, Mannheim, Germany).

### Statistics and analysis

The IBM SPSS statistics version 29 used for Data analysis. Both one-way and two-way ANOVA and the nonparametric Kruskal-Wallis H test were used to test within and between-group differences and p value < 0.05 was considered significant. A Cox proportional hazards model was used to examine the influence of dietary and lifestyle factors on the probability of developing diabetes diagnosed using the WHO cut-of-points criterion of HbA1c ≥ 6.5% at follow up after adjusting for other clinical risk indicators including age, gender, marital status, level of education, occupation, BMI, nationality and physical activity. Odds of diabetes diagnosis at follow up (HbA1c <6.5% vs. HbA1c ≥ 6.5%) stratified by different levels of physical activity are presented graphically using the Kaplan-Meier hazard curve.

## Results

**Figure 1:**
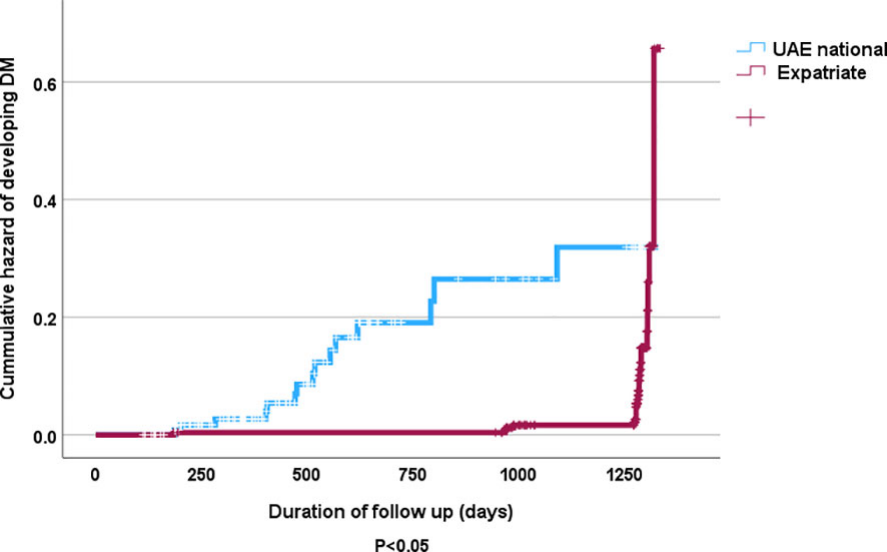
Kaplan-Meier curve of cumulative odds of developing DM (HbA1c =>6.5) in UAE nationals compared with non-UAE expatriates.

A total of 375 Community free-living locals UAE citizens subjects, 348 (93%) of them females and 253 non-locals’ expatriates [187 (73%) females] were recruited and followed up for a period of 427 ± 223 days. Using WHO cut-of-points for BMI 284 (30%) overweight, 584 (62%) obese and 69 (8%) normal body weight. **Figure 1** shows local UAE subjects and non-Gulf Arab expatriates with complete data for all variables.

**Figure 2:**
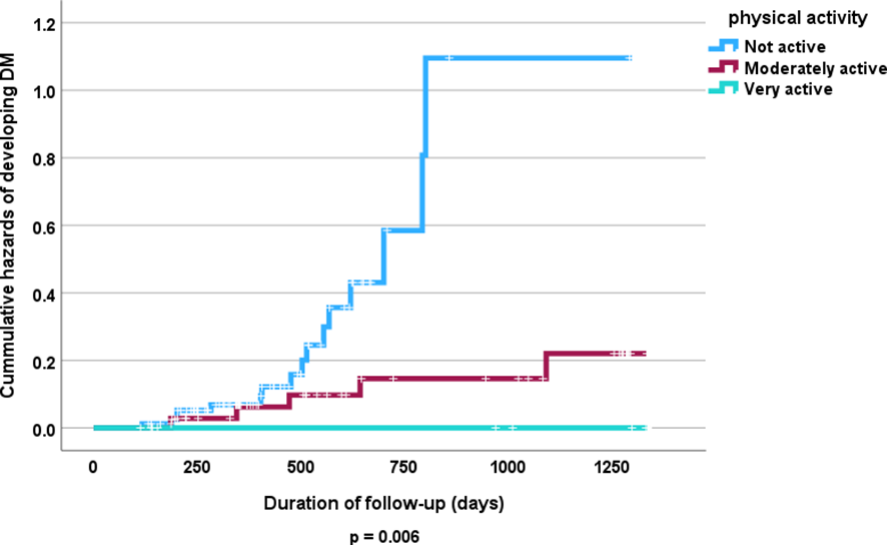
Kaplan Meier curve of cumulative odds of developing DM at follow up according to levels of physical activity in local UAE citizens.

**Figure 3:**
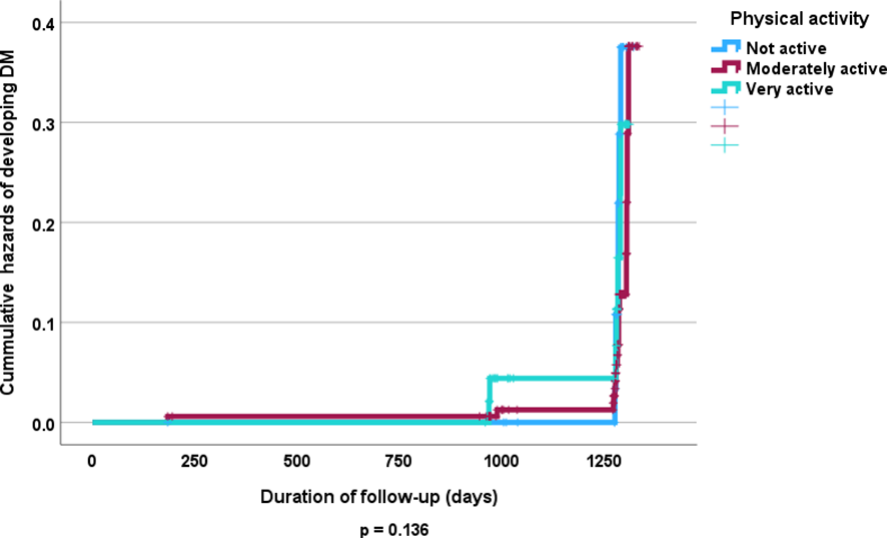
Kaplan Meier curve of cumulative odds of developing DM at follow up according to levels of physical activity in non-local expatriates.

**Figure 4:**
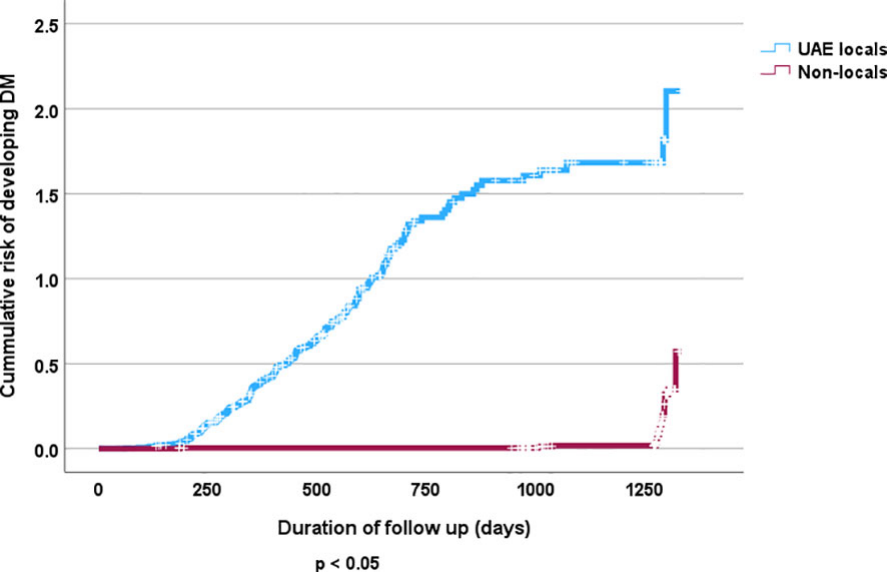
Kaplan-Meier cumulative odds of developing DM in physically inactive individuals compared with physically very active.

**Figure 5:**
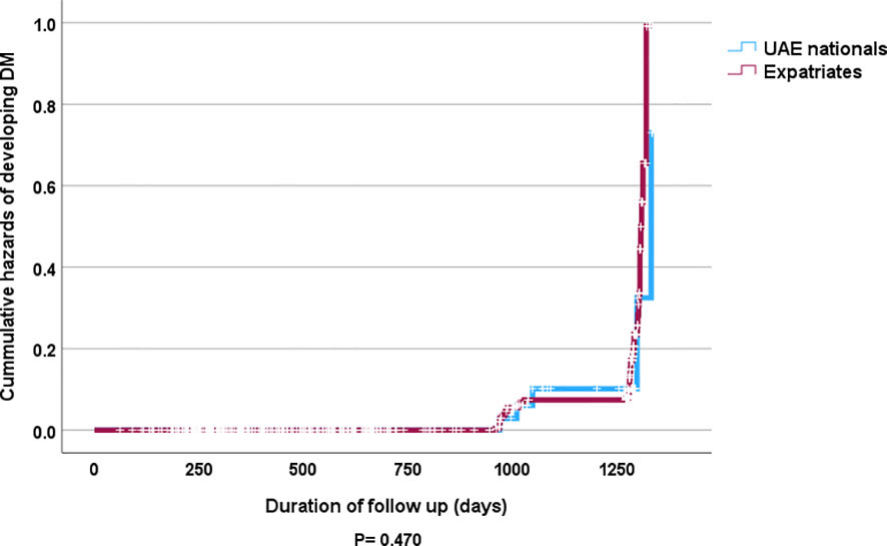
Kaplan-Meier of cumulative odds of developing DM in physically very active individuals compared with physically inactive individuals.

**Figure 6:**
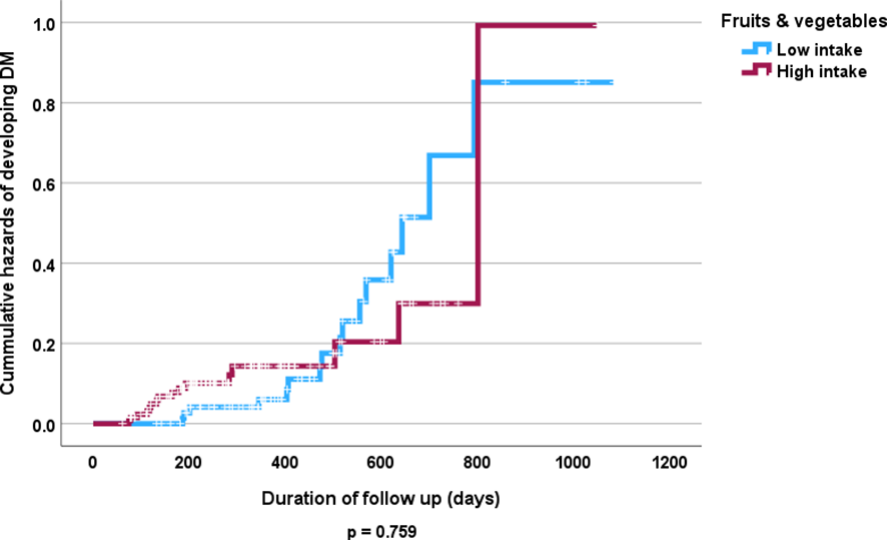
Kaplan Meier of cumulative odds of developing DM at follow according to fruits and vegetables consumption (high consumption >3.6 servings/day vs. low consumption <=3.6 servings/day).

**Figure 7:**
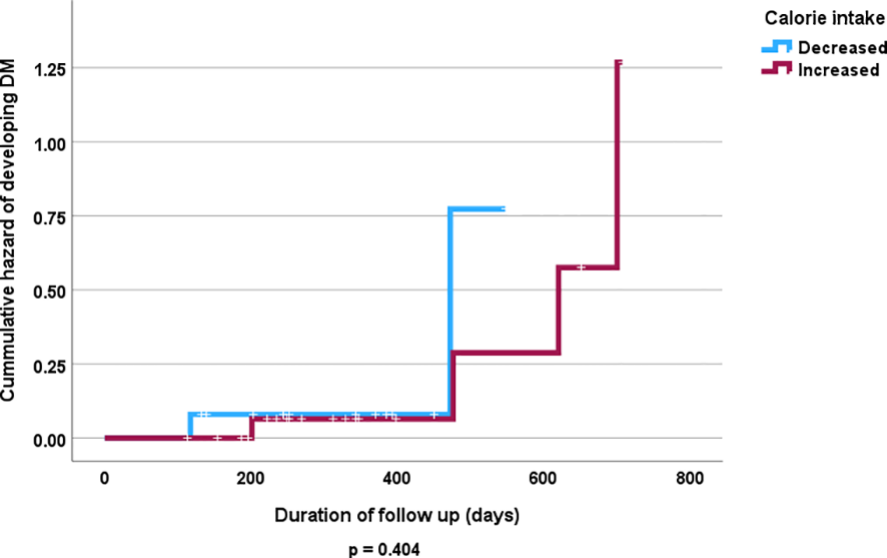
Kaplan Meier of cumulative odds of developing DM at follow according to dietary calorie intakes.

**Table 1** shows baseline characteristics including age, anthropometric, blood pressure and biochemical data of local UAE citizens compared to non-local expatriates, BMI, inflammatory marker and plasma glucose, were significantly higher with age and HDL lower in local UAE citizens compared with non-local expatriates (p<0.05). Local UAE citizens reported significantly lower levels of physical activity both at leisure or at work in comparison to non-local expatriates. The Cox proportional hazard model analysis revealed being physically inactive, local UAE citizen with increasing BMI was associated with significant increased risk of developing DM compared to non-UAE expatriates at follow up after adjusting for other prognostic indicators [Expatriates: Odd ratio (95% CI): 0.13 (0.04, 0.47); p=0.002; physically active: 0.31 (0.11, 0.90); p=0.002] (**Table 2**). **Figures 1**-**7** show the odds of diagnosis of new diabetes at follow in local UAE citizens compared to non-local expatriates stratified by different levels of physical activity and dietary intakes. For example, **Figure 1** shows the significantly increased risk of DM in UAE citizens compared with non-UAE expatriates. **Figures 2**, **3** show increased risk of DM in physical inactive UAE citizens [**Figure 2**, p <0.006] but not in non-UAE expatriates [**Figure 3**, p =0.136]. But when the group is stratified by levels of physical activity the increase in DM was only confined to physically inactive local UAE citizens [**Figure 4**, p <0.05] but not non-local expatriates [**Figure 5**, p=470]. No significant difference found in fruit and vegetables consumption or calorie intakes between subjects who develop DM compared with those who did not develop DM at follow up (**Figures 6**, **7**).

**Table 1:** Baseline clinical, anthropometric, blood pressure and biochemical data of local UAE citizens compared with non-local expatriates, mean (SD).

Variable		Locals UAE citizens (n=375)	Non-locals expatriates =253)	P value
Age (years)		35.8 (11)	41.6 (12)	0.001
Sex		348 (93)	187 (73)	0.001
Body mass index		33.7 (6)	28.95 (5)	0.001
Systolic Blood pressure (mmHg)		120 (12)	124 (16)	0.002
Diastolic blood pressure (mmHg)		73 (9)	76 (8)	0.001
Hs-CRP (mg/l)		6.73 (7.1)	3.51 (3.9)	0.001
Glucose (mmol/L)		11.1 (15)	6.0 (2)	0.001
HbA1c (%)		5.78 (0.8)	5.69 (0.8)	0.363
Total Cholesterol (mmol/L)		4.81.(83)	4.87 (.98)	0.230
Low density lipoprotein (mmol/L)		2.91 (.76)	3.29 (.87)	0.001
High density lipoprotein (mmol/L)		1.08 (.33)	1.23 (.39)	0.001
Triglycerides (mmol/L)		1.06 (.51)	1.55 (1.1)	0.001
How physically active is your leisure time	not very active	220 (67)	36 (14)	
	moderately active	102 (31)	168 (67)	
	very active	6 (2)	49 (19)	0.001

**Table 2:** The Cox’s proportional hazard analysis of the risk of developing DM in local UAE citizens compared with expatriates at follow up after adjusting for other prognostic indicators.

Variable	Significance	Hazard ratio	95.0% CI for hazard ratio	
			Lower	Upper
Age (years)	.304	1.030	.974	1.089
Sex (male/female)	.018*	10.897	1.511	78.607
Marital status (married, unmarried, divorced)	.412	1.418	.616	3.265
Occupation (employed, unemployed)	.153	1.669	.827	3.367
Level of education (primary, secondary, university)	.709	1.094	.684	1.749
BMI	.001*	1.123	1.046	1.206
Nationality group (local; non-local)	.002*	.130	.036	.472
Physical activity (not active, moderately active, very active)	.032*	.311	.107	.903

P value ≤ 0.05.

## Discussion

Overall, our results showed physical inactivity is associated with increased risk of developing DM amongst physical inactive local UAE citizens but not non-local expatriates. Coupled with low physical activity we found local UAE citizens to have had higher BMI and inflammation compared with non-local expatriates despite non-local expatriate individuals being significantly older with higher blood pressure.

When explored with BMI the influence of physical activity on the risk of DM seems to be attenuated particularly in those who already diagnosed with diabetes at baseline. This is not surprising given the fact that physical inactivity and increasing BMI have been viewed as independent variables in the development of diabetes and other obesity related risk factors (6). Another important point is the magnitude of the association with the risk of developing diabetes may be much greater with greater with BMI than physical activity. Overall, this underscores the importance of adiposity as a major risk factor for type 2 diabetes. However, physical activity still remains a significant risk factor for obesity-related diabetes but more importantly an indispensable tool for prevention and treatment.

Worldwide the prevalence of diabetes has been increasing relentlessly and at present reaching epidemic proportions (1). This is to the extent that many health care systems particularly in developing countries are no longer able to cope with the burden of diabetes treatment which is mainly affecting young and middle age working men.

The main drivers for the rise in diabetes are dietary, physical activity and genetic factors. In the Middle East and Asian countries physical inactivity for example, is a major risk factor for obesity and associated pathologies such as type 2 diabetes (12). Recent surveys report on lifestyle agree that the resident populations of the Gulf region do not get enough exercise to keep themselves healthy. This is confirmed by a systematic review of studies of current levels of physical activity levels of young residents of the UAE which revealed that almost a quarter of the young population have a total sedentary lifestyle; less than half have been mildly involved in physical activities and around a fifth practiced a moderate level of physical activity (13, 14). Furthermore, physical inactivity levels are far more prevalent than currently estimated. Studies revealed that both men overestimate their physical activity levels by a wide margin (15). In the UAE for example, where the sample of this study comes from the reasons for increased physical inactivity include the arid desert climate with high day temperature and the built environment perceived as not being conducive to walking-narrow roads, unmarked crossings besides increased traffic density and unsafe driving behavior on roads around schools and neighborhood (16, 17). But the question still remains as to why physically inactive UAE citizens have increased risk of developing DM compared to Arab expatriates living and working in the UAE. Could this difference be due to genetic factors? It has been argued that diabetes is commonest among populations who have recently been exposed to abundant high energy density food supply after centuries of famine. In such groups there may be a high prevalence of ‘thrifty genes’ that promote weight gain and hyperinsulinemia in condition of abundance. In contrast, in groups who have had good food supply for several generations the process of natural selection will have reduced the prevalence of the ‘thrifty genes’ that have become harmful in an environment of plentiful food supply (3, 7). Indeed, the Gulf countries including the UAE have been through rapid socioeconomic and social changes with urbanization over the last 50 years. At the same time changes in diet and lifestyle with increased consumption and decreased activity are most likely leading to growing epidemic of overweight/obesity, diabetes and other related cardiovascular diseases. This is more likely to occur in those who are genetically predisposed. This is particularly important in the presence of supporting postulated but not yet consistently proven genetic hypotheses. One example is the role of thrifty genes which were selected in mankind’s distant past when the supply of food was precarious; they conveyed a ‘fast insulin trigger’ & thus the ability to store food rapidly as fat (saving). They become diabetogenic in a modern setting of plentiful nutrition (18). Another specific example is the presence of Fat Mass Obesity Associated gene (FTO) which was the first variant identified to influence obesity risk (19). Single nucleotide polymorphisms have been associated with obesity risk across different age and populations (20). Studies have also demonstrated that the FTO gene may play a central role in the regulation of food intake (21). Furthermore, several studies have suggested body fatness induced by the FTO gene presence may be attenuated by increased physical activities (22). Indeed, a meta-analysis results suggested that higher physical activities attenuate the influence of FTO variation on obesity risk by 30% (23). However, the mechanism by which increased physical activity mitigates the obesity risk of the FTO gene is not well understood (24).

The recent study of the Worldwide trends in diabetes prevalence from 1990 to 2022 reported an estimated 828 million adults had diabetes, an increase of 630 million with the largest increase in South Asia, the Middle East and North Africa (5). Because the main drivers for the rise in diabetes other than physical inactivity and genetic factors another meta-analysis has reported that increasing daily intake of green leafy vegetables could significantly reduce the risk of type 2 diabetes (25). In this study we found no statistically significant effects of fruits and vegetables or calorie intake on risk of DM. However, results of the relationship between calorie intakes and incident diabetes should be interpreted with caution because this was assessed in a smaller sample both at baseline and follow up. Nevertheless, current evidence suggests low calorie intake results in weight loss which is key therapy for obesity because it can mitigate or resolve metabolic risk factors associated with obesity.

## Limitations and strength of the study

Although we have included people whose HbA1c is ≥ 6.5% but not fasting plasma glucose to define diabetes the approach is however consistent with current guidelines and practice. Another limitation of our study is the use of a validated interview questionnaire to evaluate physical activity both at baseline and at follow up as opposed to using records of physical pedometer or accelerometers. This may have introduced bias given the reported evidence that both men and women overestimate their physical activity levels by a significant amount (15). Our sample size is a large and coming from a society with the second highest prevalence of obesity related diabetes mellitus in the World. However, many did not provide a follow up blood sample, and we do acknowledge gender imbalance and the short follow-up duration. Another potential limitation is the measurement of fruits and vegetables and calorie intakes in smaller subgroups compared to the total sample size of the studied cohort. We have adjusted for important lifestyle and prognostic factors during the analysis.

## Conclusion

Physical inactivity is associated with increased risk of developing DM amongst local UAE citizens compared with non-local expatriates. In contrast risk of developing DM is no different between physically very active UAE nationals compared to non-national expatriates at follow up. Based on currently available data physical inactivity is the main driver for obesity-related diabetes will continue to increase to the extent that this epidemic will overwhelm the health-care system and make it prohibitively expensive. To protect the public, health actions including regulations are urgently needed to increase physical activity in this high-risk group. Coupled with availability of healthy food choices these actions could have enormous public health implications by mitigating obesity related adverse health effects in our community and worldwide. Simultaneously new research is needed to understand the underlying reasons genetic or otherwise for the increased risk of DM in local groups including UAE individuals and similar nations.

## Data Availability

Data is available upon request to the corresponding author.
